# Prevalence and incidence of chronic obstructive pulmonary disease in Latin America and the Caribbean: a systematic review and meta-analysis

**DOI:** 10.1186/s12890-022-02067-y

**Published:** 2022-07-16

**Authors:** Juan J. Olortegui-Rodriguez, David R. Soriano-Moreno, Alejandro Benites-Bullón, Pilar P. Pelayo-Luis, Jorge Huaringa-Marcelo

**Affiliations:** 1 Unidad de Investigación Clínica y Epidemiológica, Escuela de Medicina, Universidad Peruana Unión, Lima, Peru; 2 Facultad de Medicina Humana, Universidad Científica del Sur, Lima, Peru; 3 Hospital Nacional Arzobispo Loayza, Lima, Peru

**Keywords:** Pulmonary disease, Chronic obstructive, Epidemiology, Prevalence, Incidence, Latin America, Caribbean region

## Abstract

**Background:**

Chronic Obstructive Pulmonary Disease (COPD) remains one of the leading causes of morbidity and mortality worldwide, and its epidemiology in Latin America and the Caribbean is not well described. The aim of this study was to evaluate the prevalence and incidence of COPD in Latin America and the Caribbean.

**Methods:**

We searched systematically in Web of Science (WoS)/Core Collection, WoS/MEDLINE, WoS/Scielo, Scopus, PubMed, and Embase from 2010 to 2021. Studies assessing the prevalence and incidence of COPD according to the GOLD classification were included. The overall prevalence of COPD was calculated as a function of the general population using a random-effects model.

**Results:**

20 studies (19 cross-sectional and 1 cohort) met the inclusion criteria. The prevalence of COPD in the general population older than 35 years was 8.9%. The prevalence in men was 13.7% and in women 6.7%. The prevalence in smokers and ex-smokers was 24.3%. The incidence in the general population of COPD according to one study was 3.4% at 9 years of follow-up.

**Conclusions:**

COPD is prevalent in Latin America, especially in men and in smokers and ex-smokers. Further prevalence and incidence studies in the general population are needed, as well as health policies and strategies to address the disease.

**Supplementary Information:**

The online version contains supplementary material available at 10.1186/s12890-022-02067-y.

## Background

Chronic obstructive pulmonary disease (COPD) is a pathology with high morbidity and mortality. [1]. Approximately 300 million people have COPD globally [2], with a prevalence of approximately 12.2% [3]. This disease represents the fifth leading cause of death worldwide and it is estimated that by 2030 it will be the fourth [1]. 80% of COPD deaths occur in low- and middle-income countries [4]. Tobacco is the main cause, contributing to other co-morbidities and fatal outcomes [1]. In addition, the disease represents a high economic burden, with costs of approximately $ 5600 per patient per year [5], which increase according to the severity of the disease [6].

The diagnosis of COPD can be made based on various criteria such as symptomatology, biomarkers, ancillary questionnaires, and spirometry [7, 8]. However, the current benchmark is spirometry with an FEV1/FVC value of less than 0.7 post-bronchodilation, a criterion proposed in 2001 by the Global Initiative for Chronic Obstructive Lung Disease (GOLD) [9]. Risk factors for developing COPD include mainly passive and/or active smoking, as well as residence in highly polluted areas, exposure to biomasses, and handling of inhalation chemicals [10]. COPD has also been associated with complications such as cardiovascular disease, lung cancer, pneumonia, and even increased mortality due to SARS-CoV-2 [11, 12]. COPD patients are subjected to increased physical and psychological burden due to decreased life expectancy and performance, and associated comorbidities and respiratory symptoms (dyspnoea and exacerbations) [13, 14].

In many places or latitudes, the prevalence and/or incidence of COPD is changing. This is due to multiple factors such as increased cigarette smoking, exposure to tobacco smoke, variations in indoor and outdoor air pollution. On the other hand, screening and health service delivery programs have been established in different regions [4, 15]. In Latin America and the Caribbean, not much is known about the epidemiology of this disease. Systematic reviews have been conducted on the prevalence and incidence of COPD worldwide and in different populations; however, their results cannot be extrapolated to Latin America and the Caribbean because the included studies from this region are few or non-existent [3], or are not uniform in the diagnostic criteria for COPD [16]. A previous systematic review has also been conducted in Latin America and the Caribbean [17]. However, we believe that an update is needed, given the fact that 10 years have passed. Therefore, this systematic review aimed to identify the prevalence and incidence of COPD in the Latin American and Caribbean populations.

## Methods

We performed a systematic review following the Preferred Reporting Items for Systematic Reviews and Meta-Analysis (PRISMA) guidelines 2020 [18]. The study protocol has been registered at PROSPERO, number CRD42021233807.

### Eligibility criteria

We included cross-sectional observational and cohort studies reporting prevalence or incidence of COPD in different settings (general population, primary care centers, or hospitals) conducted in Latin America and the Caribbean since 2010. Following the protocol, we considered studies that assessed COPD with the classification of airflow limitation severity (GOLD: post-bronchodilator FEV1/FVC ratio < 0.70) [9]. Studies with fewer than 30 patients, manuscripts not available in full text, and duplicate populations were excluded. In the case of duplicate populations, the most complete study was included.

### Literature search and study selection

A systematic search was conducted in six databases: Web of Science (WoS)/Core Collection, WoS/MEDLINE, WoS/Scielo, Scopus, PubMed, and Embase between January 1, 2010, and 23 March 2021. No language restrictions were applied. The full search for each database is available in Additional file 1: Material S1. We also reviewed the reference list of all included studies and previous systematic reviews for additional eligible studies.

The identified references were exported to Rayyan software where duplicates were manually removed. Subsequently, the authors (ABB, JJOR, PPL) screened the articles by titles and abstracts to identify potentially relevant articles for inclusion. Selected studies were then reviewed at the full text (ABB, JJOR). These processes were conducted independently and discrepancies were resolved in meetings with all authors to decide whether the study was included.

### Data extraction

Two authors (ABB, JJOR) independently extracted the following data of interest using a Microsoft Excel sheet: author, year of publication, study design, country, setting, smoking status, sample size, age, sex, disease prevalence/incidence. Discrepancies were resolved in a meeting.

### Risk of bias

Three authors (ABB, JJOR, PPL) independently assessed the methodological quality of prevalence and incidence studies using the Joanna Briggs Institute Critical Appraisal Tool [19]. Another author (DRSM) resolved discrepancies at this stage. This scale has 9 items with possible responses of "Yes", "No" and "Unclear". The quality score presented in Table 1 was found by considering "Yes" as one point and "No" and "Unclear" as zero points, i.e. the higher the score the lower the risk of bias.

**Table 1:** Characteristics of included studies assessing the prevalence of chronic obstructive pulmonary disease in Latin America and the Caribbean (n = 19)

Study id	Country	Setting	Smoking status	Sample size	Age (years) mean ± SD	Male sex (%)	COPD prevalence (%)	Quality score (Max. 9)
Romero-Lopez – [34]	Mexico	Hospital	Non-smokers, ex-smokers or smokers	66	NR	97.0	7.6	5
Bastidas – [35]	Colombia	Hospital	Non-smokers, ex-smokers or smokers	1599	65.3 ± 12.0	43.9	21.3	6
Conyette – [36]	Trinidad and Tobago	General population	Non-smokers, ex-smokers or smokers	1104	NR	40.1	9.5	9
Ramirez-Venegas – [30]	Mexico	General population	Non-smokers, ex-smokers or smokers	969	49.8 ± 11.1	0.0	2.5	7
Echazarreta – [31]	Argentina	General population	Non-smokers, ex-smokers or smokers	3469	58.8 ± 11.6	42.1	14.5	8
Siddharthan – [32]	Argentina	General population	Non-smokers, ex-smokers or smokers	2335	58.2 ± 8.0	60.5	10.7	7
	Chile			1038	59.2 ± 8.5	54.7	5.2	
	Uruguay			851	59.6 ± 8.5	61.7	9.3	
Vieira – [33]	Brazil	General population	Smokers	183	54.5 ± 9.2	42.6	19.7	6
Karloh – [37]	Brazil	General population	Non-smokers, ex-smokers or smokers	1057	58.0	40.1	8.7	9
Sobrino – [26]	Argentina	General population	Non-smokers, ex-smokers or smokers	2352	NR	NR	9.9	9
	Chile			1063			6.4	
	Uruguay			939			11.0	
López—[25]	Argentina	Primary care	Ex-smokers or smokers	446	NR	NR	29.6	6
	Colombia			326			26.4	
	Venezuela			665			11.0	
	Uruguay			103			17.5	
Jaganath – [22]	Peru	General population	Non-smokers, ex-smokers or smokers	2957	55.3 ± 12.4	49.3	6.0	9
Rabahi—[23]	Brazil	Primary care	Ex-smokers or smokers	316	NR	NR	16.1	3
Sansores—[24]	Mexico	General population	Ex-smokers or smokers	2961	51.4 ± 10.7	49.5	11.1	6
Moreira – [20]	Brazil	Hospital	Non-smokers exposed to wood stove smoke	160	64.6 ± 10.1	0.0	26.9	3
Orduz – [21]	Colombia	General population	Non-smokers	578	NR	NR	6.7	5
Queiroz – [27]	Brazil	Primary care	Ex-smokers or smokers	200	65.0 ± 10.4	NR	31.5	6
Laniado-Laborin – [28]	Mexico	General population	Ex-smokers or smokers	2293	57.6 ± 12.2	39.8	20.6	8
Chatkin – [38]	Brazil	Hospital	Smokers	294	55.3 ± 10.9	44.2	59.2	5
Lopez-[29]	Brazil, Chile, Mexico, Uruguay, Venezuela	General population	Non-smokers, ex-smokers or smokers	5314	56.3 ± 0.2	39.6	14.3	8

*NR* Not reported, *COPD* Chronic obstructive pulmonary disease

### Statistical analyses

We performed the analyses with STATA V16.0 software. We calculated pooled COPD prevalence in the general population, using a random-effects model, with their 95% confidence intervals using the exact method. We used the Freeman-Tukey Double Arcsine transformation to stabilize variances. The criteria for including studies in the main meta-analysis were: studies conducted in patients in the community and including smokers, ex-smokers, and non-smokers. To assess heterogeneity and its sources, we used the I^2^ test and performed subgroup analyses according to sex, country, and risk of bias. We also performed a sensitivity analysis to assess the variation in prevalence when excluding each article from the meta-analysis. Publication bias was assessed with Egger's statistic considering a *p* < 0.05 as statistically significant. On the other hand, we assessed prevalences according to smoking status (non-smokers, ex-smokers, and smokers) and according to study setting (community, primary care center, and hospital).

## Results

### Selection of studies

**Fig. 1:**
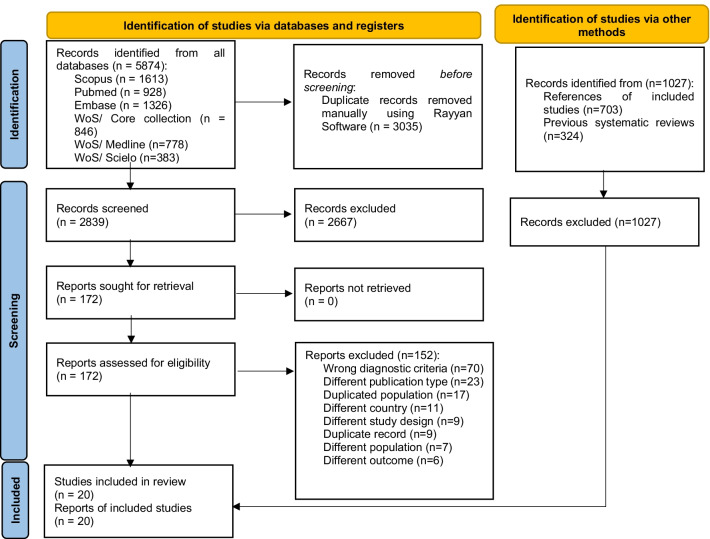
Flow diagram summarizing the process of literature search and selection

The systematic search identified 5874 studies, with 2839 studies remaining after the removal of duplicates. Studies were screened by title and abstract, and 172 were selected for full-text review. In the full-text review, 151 studies were excluded because they did not meet the eligibility criteria, leaving 20 studies, which were included in the full-text review [20–39] were included in the review (Fig. 1). The excluded full-text studies and reasons for exclusion are shown in Additional file 1: Material S2.

### Characteristics of the studies included

Of the 20 studies that met the eligibility criteria, 19 were cross-sectional studies (Table 1) and only one was longitudinal (cohort study). Of the cross-sectional studies, the number of participants was 33 637 and ranged from 66 to 5314. All studies included participants older than 35 years. The mean age ranged from 49.8 to 65.3. The countries in which the studies were conducted were Brazil [20, 23, 27, 29, 33, 37–39], Mexico [24, 28–30, 34], Argentina [25, 26, 31, 32] Uruguay [25, 26, 29, 32], Chile [26, 29, 32], Colombia [21, 25, 35], Peru [22], and Trinidad and Tobago [36]. Thirteen studies were conducted in the general population [21, 22, 24, 26, 28–33, 36, 37, 39] four in hospitals [20, 34, 35, 38] and three in primary care centers [23, 25, 27]. Concerning the type of population, both Moreira et al. and Ramirez-Venegas et al. were studies conducted only in the female population [20, 30]; Romero-Lopez et al. conducted the study in people with HIV [34]. Three studies were conducted in an older adult population [20, 27, 35]. Bastidas et al. evaluated patients scheduled for spirometry [35]. Regarding smoking status, most studies included smokers, ex-smokers, and non-smokers. Seven studies had a high population prevalence of smokers or ex-smokers [23, 24, 27–29, 33, 38]. Moreira et al. included non-smoking women exposed to wood smoke [20]. The study by Orduz et al. was conducted on non-smokers. [21].

Regarding the cohort study, it was conducted in the general population in Brazil with a follow-up of 9 years [39]. The number of participants was 594, all of whom were aged 40 years or older. The study included non-smokers, former smokers, and current smokers (Additional file 1: Material S3).

### Prevalence of COPD in Latin America and the Caribbean

**Fig. 2:**
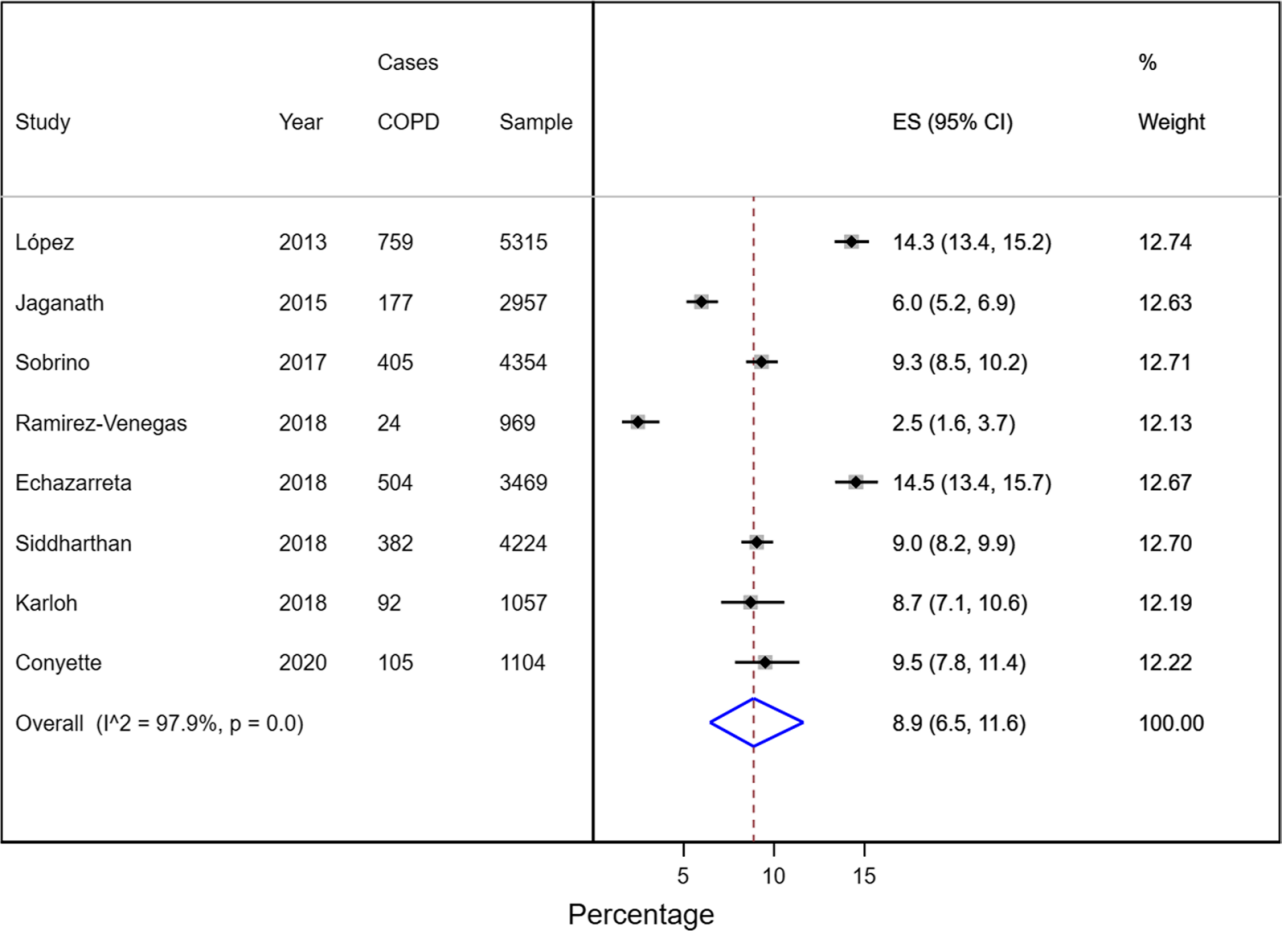
Prevalence of chronic obstructive pulmonary disease in the general population of Latin America and the Caribbean

Of the 19 cross-sectional studies, 8 (n = 23,449) met the criteria to be included in the main meta-analysis [22, 26, 29–32, 36, 37]. The prevalence of COPD in the general population was 8.9% (95% CI: 66–116; I2^2^: 97.9%) with a range of 2.5 to 14.5% (Fig. 2). In the included studies, all participants were older than 35 years with a mean age between 39.6 and 58.8. Most of the included studies were in both sexes, with some predominance of the male population (approximately 60% male and 40% female).

### Sub-group analysis

**Fig. 3:**
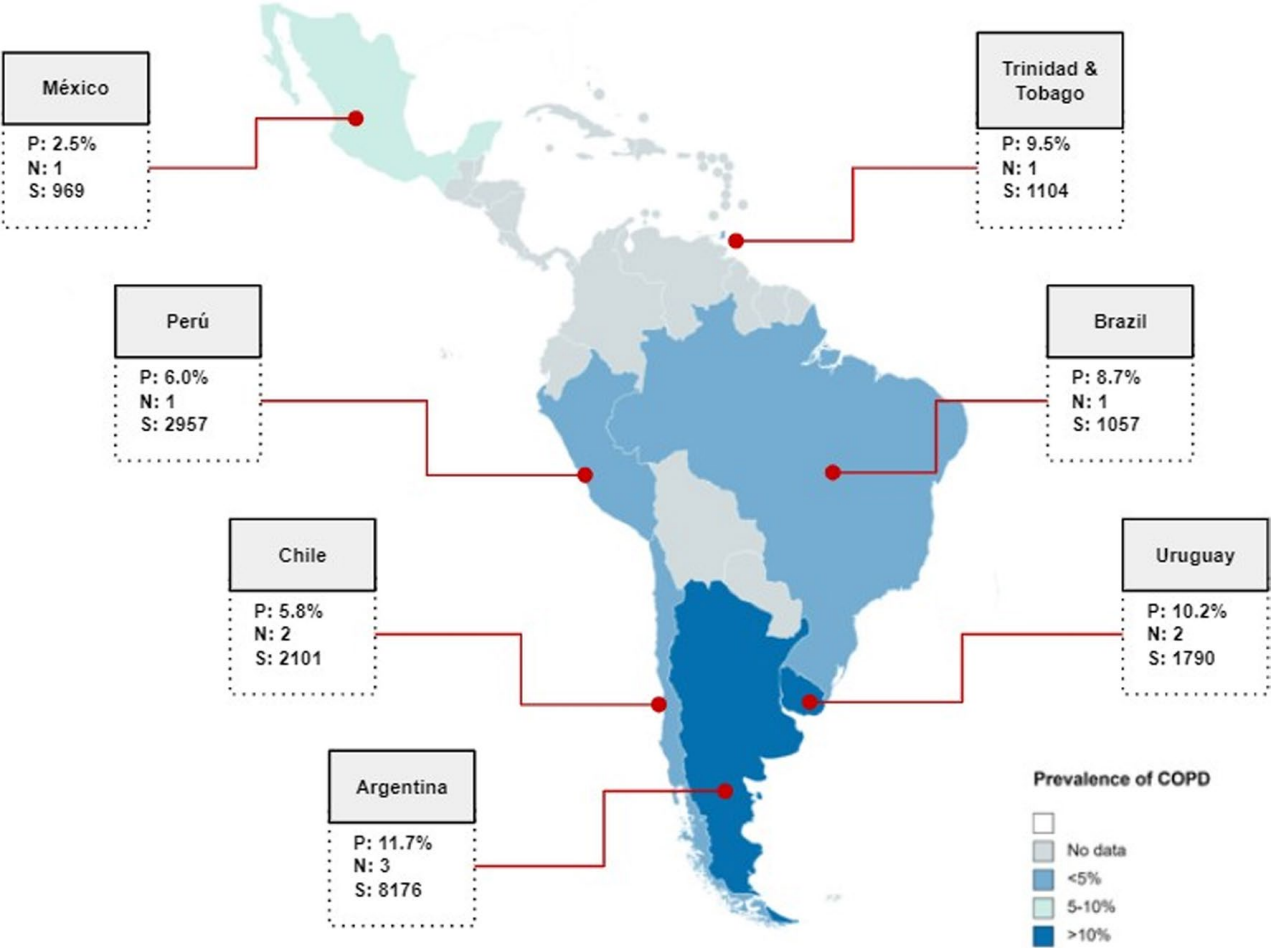
Prevalence of chronic obstructive pulmonary disease in the general population of Latin America and the Caribbean countries

In addition, COPD prevalence was assessed according to sex, country, and risk of bias. With respect to sex, COPD prevalence was higher in men (13.7%; 95% CI: 9.7–18.3; IQ^2^: 96.4%) than in women (6.7%; 95% CI: 4.0–10.0; IQ^2^: 97.4%) (Additional file 1: Material S4). Regarding country, the highest prevalence was in Argentina (11.7%; 95% CI: 8.9–14.7), while the lowest was in Mexico (2.5%; 95% CI: 1.6–3.7) (Fig. 3 and Additional file 1: Material S5). In terms of risk of bias, the studies with the highest methodological quality gave a prevalence of 8.3% (95% CI: 6.4–10.3; I^2^: 90.5%) (Additional file 1: Material S6).

In addition, studies were found that evaluated patients who were smokers and ex-smokers and in other settings. Concerning smoking status, the prevalence of COPD was higher in smokers and ex-smokers (24.3%; 95% CI: 16. 5–33.1; I^2^: 98.4%), ranging from 11.1 to 59.2% (Additional file 1: Material S7). Additionally, we found that prevalences in studies conducted in primary care centers (21.9%; 95% CI: 15.6–29.0) and in hospitals (27.3%; 95% CI: 10.2–48.8; I^2^: 98.3%) were higher than in the general population (Additional file 1: Material S8).

### Publication bias

Using Egger's test, no publication bias (p = 0.196) was found in the meta-analysis.

### Incidence of COPD in Latin America and the Caribbean

On the other hand, a cohort study found a cumulative incidence of COPD of 3.4% at 9 years follow-up. Of these cases, 40% were non-smokers, 35% smokers, and 25% ex-smokers [39] (Additional file 1: Material S3).

### Risk of bias

When assessing the risk of bias, more than 90% of the studies met the items of adequate sample size, subjects, and setting described in detail, use of validated methods for the identification of COPD, and reliably measured condition in all participants. On the other hand, a large proportion did not meet the items of the appropriate sampling frame, sampling, and statistical analysis. The overall assessment score is given in Table 1 and detailed in the Additional file 1: Material S9.

## Discussion

In this systematic review we found that the prevalence of COPD in Latin American patients was 8.9% (95% CI: 6.5–11.6; I2: 97.9%), while the cumulative incidence, obtained from 1 study, was 3.4% at 9 years.

In this systematic review, we aimed to estimate the prevalence of COPD in Latin America and the Caribbean [9]. We found that the prevalence of COPD according to GOLD criteria in the general population of the eight meta-analyzed studies (8.9%; 95% CI: 6.5–11.6; I2: 97.9%), was similar to that reported in a global systematic review (12.2%; 95% CI: 10.9–13.4; I2: 29.8%) [3] and to a Latin American and Caribbean systematic review whose search date was 2012 (13.4%; 95% CI: 10.1–17.1; I2: 94.9%) [17]. This similarity may be because the previous reviews were conducted in the general population and used the GOLD criteria. These figures show that the prevalence of COPD is maintained even though smoking has decreased in recent years [40], possibly due to other risk factors such as exposure to environmental pollution, biomasses, etc. [41].

On the other hand, when we evaluated prevalence by country, we found considerable variability. This can be explained by differences in biomass exposure, smoking levels, industrialization, genetic factors (e.g. alpha-1 antitrypsin deficiency) and the burden of other predisposing factors such as tuberculosis or asthma [22, 42, 43]. Compared to the present study, the systematic review by Ciapponi et al. (search date: 2012) reported higher prevalences in the countries of Mexico (7.8%), Brazil (15.2 to 15.8%), Chile (16.9%), and Uruguay (19.7%). Considering that the populations are from the same region, these differences may be attributed to the decline in tobacco use in these countries in recent years [44]. Public health strategies could have helped to reduce tobacco use [45].

Regarding sex, we found that the prevalence in men was twice that of women (7% more), although there is overlap in the confidence intervals. Similarly, the global systematic review of Varmaghani et al. [3] and the Latin American and Caribbean review by Ciapponi et al. [17] found a difference of 5% and 7.8% in favor of men, respectively. This indicates that, despite geographical and cultural differences, both in LATAM and globally, the prevalence of COPD is higher in men than in women. This finding is in agreement with previous literature [46] and could be explained by higher smoking habits in men than in women. [47].

In addition, we observed that the prevalence of COPD among non-smokers was lower among current or former smokers, which is explained by the fact that smoking is the main risk factor for COPD [48]. COPD in non-smoking patients could be due to indoor and outdoor pollution, occupational exposures (agriculture, dust…), treated pulmonary tuberculosis, chronic asthma, low socioeconomic status, and poor nutrition [49].

We also found that the prevalences of studies conducted in primary care centers and hospitals were higher compared to those studies conducted in the community. This is possibly because primary care centers and hospitals care for symptomatic individuals and individuals with risk factors for the disease [50].

In order to reduce the prevalence of COPD in this region, we suggest the following: to create or improve policies and legislation regarding tobacco and its derivates; to create or improve screening programs across the health systems; and to implement or strengthen the general knowledge of non-transmittable diseases, like COPD, at schools with educational programs [51].

### Limitations of the studies

The statistical heterogeneity between studies was high and did not decrease in the subgroup analyses, possibly because the confidence intervals of the included studies are narrow and do not overlap with each other. Regarding the risk of bias, studies included in the general population meta-analysis were at low risk. We recommend that future studies be more detailed in describing the population, and present COPD prevalence by smoking status, age and sex. We also recommend that they use the GOLD criteria and report that they are using them [9], as more than 70 studies were excluded for that reason in the selection process. In addition, further well-designed studies that include people over 40 years of age and that assess the incidence and prevalence in other countries are needed to understand the epidemiological behavior of COPD in the region.

### Limitations and strengths

Our systematic review has some limitations. First, we did not search the grey literature, so we believe that there may be studies in non-indexed journals or repositories that were not included in this review. In that sense, we did not find the information available for more than half of the countries in the region, so the external validity of our results should be interpreted with caution. The statistical heterogeneity of the studies included in the quantitative synthesis was high.

On the other hand, our review has several strengths. We conducted a comprehensive systematic search of global as well as regional databases and reviewed citations of included studies. We covered a period for which COPD prevalence had not been reported in previous reviews. The same diagnostic criteria for COPD were taken into consideration to reduce clinical heterogeneity among the included studies. Finally, the overall and subgroup analysis was performed taking into consideration the corresponding clinical context as well as the methodological quality of the included studies.

## Conclusion

The prevalence of COPD in the general population in Latin America and the Caribbean was 8.9%. Prevalence was higher in men, in patients who were smokers and/or ex-smokers, and in studies conducted in hospitals and primary care centers. Only one study reported that the cumulative incidence of COPD at 9 years of follow-up was 3.4%. More incidence and prevalence studies in the general population of other countries are needed. We also suggest that health strategies and policies for early detection and prevention of COPD should be considered, especially in populations with the highest prevalence.

## Supplementary Information

**Additional file 1**. Supplementary materials S1–9.

## Data Availability

All data generated or analysed during this study are included in this published article and its Additional file 1.

## Abbreviations

COPD: Chronic obstructive pulmonary disease
GOLD: Global initiative for chronic obstructive lung disease
PRISMA: Preferred reporting items for systematic reviews and meta-analysis
