# Supplementary material for: Prevalence and incidence of chronic obstructive pulmonary disease in Latin America and the Caribbean: a systematic review and meta-analysis
Source: BMC Pulm Med. 2022 Jul 16;22:273. doi: 10.1186/s12890-022-02067-y (PMC9288210; doi:10.1186/s12890-022-02067-y)
Supplement: Supplementary file 1 — Additional file 1. Supplementary materials S1–9. [file 12890_2022_2067_MOESM1_ESM.docx]

**Supplementary material**

## Supplementary material 1. Search strategy

**Filter:** 2010-2021

| Search strategy | Date of Search | Results |
| --- | --- | --- |
| **Pubmed** |  |  |
| prevalence[mesh] OR incidence[mesh] OR Observational Study[Publication Type] OR Observational studies as topic[mesh] OR Cross-Sectional Studies[mesh] OR Cohort Studies[mesh] OR Longitudinal Studies[mesh] OR prevalenc*[tiab] OR incidenc*[tiab] OR “cross-sectional”[tiab] OR “cross sectional”[tiab] OR “cohort”[tiab] OR longitudinal*[tiab] OR transvers*[tiab] OR prospective*[tiab] OR retrospective*[tiab] OR “follow-up*”[tiab] | 23/03 | 928 |
| Latin America[Mh] OR "Latin America"[tiab] OR Caribbean Region[Mh] OR "Caribbean Region"[tiab] OR South America [Mh] OR "South America”[tiab] OR Indians, South American [Mh] OR Hispanoamerica*[tiab] OR Iberoamerica*[tiab] OR Panamerican*[tiab] OR Argentina[Mh] OR Argentina[tiab] OR Argentin*[ad] OR Bolivia[Mh] OR Bolivia[tiab] OR Bolivia[ad] OR Brazil[Mh] OR Brazil[tiab] OR Brazil*[ad] OR Brasil*[ad] OR Chile[Mh] OR Chile[tiab] OR Colombia[Mh] OR Colombia[tiab] OR Colombia[ad] OR Costa rica[Mh] OR Costa rica[tiab] OR Costa Ric*[ad] OR Cuba[Mh] OR Cuba[tiab] OR Ecuador[Mh] OR Ecuador[tiab] OR Ecuador*[ad] OR El salvador[Mh] OR "El salvador"[tiab] OR "El salvador"[ad] OR Guatemala[Mh] OR Guatemala[tiab] OR Guatemala[ad] OR Haiti[Mh] OR Haiti[tiab] OR Honduras[Mh] OR Honduras[tiab] OR Mexico[Mh] OR Mexico[tiab] or Mexico[ad] or Mejico[ad] OR Nicaragua[Mh] OR Nicaragua[tiab] OR Panama[Mh] OR Panama[tiab] OR Paraguay[Mh] OR Paraguay[tiab] OR Paraguay[ad] OR Peru[Mh] OR Peru[tiab] OR Peru*[ad] OR Puerto Rico[Mh] OR "Puerto Rico"[tiab] OR "Puerto Rico"[ad] OR Dominican Republic[Mh] OR "Dominican Republic"[tiab] OR "Dominican Republic"[ad] OR Uruguay[Mh] OR Uruguay[tiab] OR Uruguay[ad] OR Venezuela [Mh] OR Venezuela [tiab] OR Venezuela [ad] OR Suriname[Mh] OR Suriname[tiab] OR Surinam*[ad] OR Guiana*[tiab] OR Guiana*[ad] OR Guyan*[tiab] OR Guyan*[ad] |  |  |
| “Pulmonary Disease, Chronic Obstructive”[MH] OR “chronic obstructive pulmonary dis*” [TIAB] OR “chronic obstructive pulmonary dis*” [OT] OR “Chronic Obstructive Lung Dis*” [TIAB] OR “Chronic Obstructive Lung Dis*” [OT] OR “Chronic Obstructive Airway Dis*” [TIAB] OR “Chronic Obstructive Airway Dis*” [OT] OR “chronic obstructive respiratory dis*” [TIAB] OR “chronic obstructive respiratory dis*” [OT] OR “lung chronic obstructive dis*” [TIAB] OR “lung chronic obstructive dis*” [OT] OR “obstructive respiratory tract dis*” [TIAB] OR “obstructive respiratory tract dis*” [OT] OR COPD[ TIAB] OR COPD[OT] OR “chronic obstructive bronchitis” [TIAB] OR “chronic obstructive bronchitis” [OT] OR “chronic obstructive bronchopulmonary dis* ” [TIAB] OR “chronic obstructive bronchopulmonary dis* ” [OT] OR “Chronic Airflow Obstruction*” [TIAB] OR “Chronic Airflow Obstruction*” [OT] OR “chronic airway obstruction*” [TIAB] OR “chronic airway obstruction*” [OT] OR Pulmonary Emphysem [MH] OR emphysem* [TIAB] OR emphysem* [OT] OR “Bronchitis, Chronic” [MH] OR “Chronic bronch*” [TIAB] OR “Chronic bronch*” [OT] OR “Bronchitis chronic*” [TIAB] OR “Bronchitis chronic*” [OT]  #1 AND #2 AND #3 |  |  |
| **Scopus** | | |
| TITLE-ABS-KEY(Prevalen* OR incidenc* OR “cross$sectional” OR cohort OR longitudinal* OR frequenc* OR transvers* OR prospective* OR retrospective* OR “follow-up*” OR “follow up*”) OR TITLE-ABS-KEY (observational W/2 stud*) |  | 1613 |
| AFFILCOUNTRY ( argentina OR bolivia OR brazil OR brasil OR colombia OR chile OR ecuador OR guyana OR "french Guiana" OR paraguay OR peru OR suriname OR uruguay OR venezuela OR belize OR "costa rica" OR "el Salvador" OR guatemala OR honduras OR nicaragua OR panama OR mexico OR mejico OR cuba OR "dominican republic" OR haiti OR jamaica OR "Puerto rico" OR "trinidad and tobago" OR barbados OR guadeloupe OR grenada OR martinique OR bermuda OR bahamas ) |  |  |
| TITLE-ABS-KEY(“lung chronic obstructive dis*” OR COPD OR “chronic airway obstruction*” OR emphysem*) OR TITLE-ABS-KEY(“chronic obstructive” W/2 (“pulmonary dis*” OR “lung dis*” OR airway OR “respiratory dis*” OR “bronch*” OR “bronchopulmonary dis*”)) OR TITLE-ABS-KEY(Chronic W/1 bronchitis) |  |  |
| **Embase** | | |
| (Incidence OR prevalence OR ‘cross-sectional study’ OR ‘cohort analysis’ OR ‘frequency analysis’ OR ‘prospective study’ OR ‘retrospective study’ OR ‘follow up’ OR ‘observational study’)/exp OR (Prevalen* OR incidenc* OR “cross-sectional” OR “cross sectional” OR cohort OR longitudinal* OR frequenc* OR transvers* OR prospective* OR retrospective* OR “follow-up*” OR “follow up*”):ti,ab,kw OR (observational NEAR/2 stud*):ti,ab,kw |  | 1326 |
| 'argentina':ca OR 'bolivia':ca OR 'brazil':ca OR brasil:ca OR 'colombia':ca OR 'chile':ca OR 'ecuador':ca OR 'guyana':ca OR 'french guiana':ca OR 'paraguay':ca OR 'peru':ca OR 'suriname':ca OR 'uruguay':ca OR 'venezuela':ca OR 'belize':ca OR 'costa rica':ca OR 'el salvador':ca OR 'guatemala':ca OR 'honduras':ca OR 'nicaragua':ca OR 'panama':ca OR 'mexico':ca OR mejico:ca OR 'cuba':ca OR 'dominican republic':ca OR 'haiti':ca OR 'jamaica':ca OR 'puerto rico':ca OR 'trinidad and tobago':ca OR 'barbados':ca OR 'guadeloupe':ca OR 'grenada':ca OR 'martinique':ca OR 'bermuda':ca OR 'bahamas':ca |  |  |
| 'chronic obstructive lung disease':de OR 'chronic airflow obstruction':tn,ti,ab OR 'chronic airway obstruction':tn,ti,ab OR 'chronic obstructive bronchitis':tn,ti,ab OR 'chronic obstructive bronchopulmonary disease':tn,ti,ab OR 'chronic obstructive lung disease':tn,ti,ab OR 'chronic obstructive lung disorder':tn,ti,ab OR 'chronic obstructive pulmonary disease':tn,ti,ab OR 'chronic obstructive pulmonary disorder':tn,ti,ab OR 'chronic obstructive respiratory disease':tn,ti,ab OR 'copd':tn,ti,ab OR 'lung chronic obstructive disease':tn,ti,ab OR 'lung disease, chronic obstructive':tn,ti,ab OR 'lung diseases, obstructive':tn,ti,ab OR 'obstructive lung disease':tn,ti,ab OR 'obstructive lung disease, chronic':tn,ti,ab OR 'obstructive lung diseases':tn,ti,ab OR 'obstructive pulmonary disease':tn,ti,ab OR 'obstructive respiratory disease':tn,ti,ab OR 'obstructive respiratory tract disease':tn,ti,ab OR 'pulmonary disease, chronic obstructive':tn,ti,ab OR 'pulmonary disorder, chronic obstructive':tn,ti,ab |  |  |
| **WoS** | | |
| TS=(Prevalen* OR incidenc* OR “cross$sectional” OR cohort OR longitudinal* OR frequenc* OR transvers* OR prospective* OR retrospective* OR “follow$up*”) OR TS=(observational NEAR/2 stud*) |  | 846 |
| CU= (argentina OR bolivia OR brazil OR BRASIL OR colombia OR chile OR ecuador OR guyana OR "french Guiana" OR paraguay OR peru OR suriname OR uruguay OR venezuela OR belize OR "costa rica" OR "el Salvador" OR guatemala OR honduras OR nicaragua OR panama OR mexico OR mejico OR cuba OR "dominican republic" OR haiti OR jamaica OR "Puerto rico" OR "trinidad and tobago" OR barbados OR guadeloupe OR grenada OR martinique OR bermuda OR bahamas) |  |  |
| TS=(“lung chronic obstructive dis*” OR COPD OR “chronic airway obstruction*” OR emphysem*) OR TS=(“chronic obstructive” NEAR/2 (“pulmonary dis*” OR “lung dis*” OR airway OR “respiratory dis*” OR “bronchitis” OR “bronchopulmonary dis*”)) OR TS=(Chronic NEAR/1 bronchitis) |  |  |
| **Scielo (WoS)** | | |
| TS=(Prevalen* OR incidenc* OR “cross$sectional” OR cohort OR longitudinal* OR frequenc* OR transvers* OR prospective* OR retrospective* OR “follow$up*”) OR TS=(observational NEAR/2 stud*) |  | 383 |
| CU= (argentina OR bolivia OR brazil OR BRASIL OR colombia OR chile OR ecuador OR guyana OR "french Guiana" OR paraguay OR peru OR suriname OR uruguay OR venezuela OR belize OR "costa rica" OR "el Salvador" OR guatemala OR honduras OR nicaragua OR panama OR mexico OR mejico OR cuba OR "dominican republic" OR haiti OR jamaica OR "Puerto rico" OR "trinidad and tobago" OR barbados OR guadeloupe OR grenada OR martinique OR bermuda OR bahamas) OR AD= (argentina OR bolivia OR brazil OR BRASIL OR colombia OR chile OR ecuador OR guyana OR "french Guiana" OR paraguay OR peru OR suriname OR uruguay OR venezuela OR belize OR "costa rica" OR "el Salvador" OR guatemala OR honduras OR nicaragua OR panama OR mexico OR mejico OR cuba OR "dominican republic" OR haiti OR jamaica OR "Puerto rico" OR "trinidad and tobago" OR barbados OR guadeloupe OR grenada OR martinique OR bermuda OR bahamas) |  |  |
| TS=(“lung chronic obstructive dis*” OR COPD OR “chronic airway obstruction*” OR emphysem*) OR TS=(“chronic obstructive” NEAR/2 (“pulmonary dis*” OR “lung dis*” OR airway OR “respiratory dis*” OR “bronchitis” OR “bronchopulmonary dis*”)) OR TS=(Chronic NEAR/1 bronchitis) |  |  |
| **Medline** |  |  |
| TS=(Prevalen* OR incidenc* OR “cross$sectional” OR cohort OR longitudinal* OR frequenc* OR transvers* OR prospective* OR retrospective* OR “follow$up*”) OR TS=(observational NEAR/2 stud*) |  | 778 |
| AD= (argentina OR bolivia OR brazil OR BRASIL OR colombia OR chile OR ecuador OR guyana OR "french Guiana" OR paraguay OR peru OR suriname OR uruguay OR venezuela OR belize OR "costa rica" OR "el Salvador" OR guatemala OR honduras OR nicaragua OR panama OR mexico OR mejico OR cuba OR "dominican republic" OR haiti OR jamaica OR "Puerto rico" OR "trinidad and tobago" OR barbados OR guadeloupe OR grenada OR martinique OR bermuda OR bahamas) |  |  |
| TS=(“lung chronic obstructive dis*” OR COPD OR “chronic airway obstruction*” OR emphysem*) OR TS=(“chronic obstructive” NEAR/2 (“pulmonary dis*” OR “lung dis*” OR airway OR “respiratory dis*” OR “bronchitis” OR “bronchopulmonary dis*”)) OR TS=(Chronic NEAR/1 bronchitis) |  |  |
| ENCABEZADO MeSH: (Pulmonary Disease Chronic Obstructive)  MH=(Pulmonary Disease Chronic Obstructive) |  |  |

## Supplementary material 2. Excluded studies reviewed in full text

| Author - Year | Title | Reason for exclusion |
| --- | --- | --- |
| Lange - 2015 | Lung-Function Trajectories Leading to Chronic Obstructive Pulmonary Disease. | Different country |
| Sood - 2014 | Spirometry and health status worsen with weight gain in obese smokers but improve in normal-weight smokers. | Different country |
| Marin-Sanchez - 2020 | [Basic clinical characteristics in the first 100 fatal cases of COVID-19 in Colombia]. | Different diagnostic criteria |
| Souza - 2020 | Prevalence and factors associated with respiratory diseases and diarrhea in recyclable material cooperative workers in the city of Sao Paulo, Brazil: a cross-sectional study, 2013. | Different diagnostic criteria |
| Ahumada - 2013 | Prevalence of abnormal lung volumes, DLCO and chest HRCT in smokers with normal spirometry | Different publication type |
| Lamprecht - 2013 | Heterogeneity in prevalence and underdiagnosis of COPD: Results from BOLD, EPI-SCAN, PLATINO, and PREPOCOL | Different diagnostic criteria |
| Thorington - 2011 | Prevalence of chronic obstructive pulmonary disease among stable chronic disease subjects in primary care in Trinidad, West Indies. | Different diagnostic criteria |
| Sood - 2010 | Wood smoke exposure and gene promoter methylation are associated with increased risk for COPD in smokers. | Different country |
| Leal - 2020 | Epidemiology and burden of chronic respiratory diseases in Brazil from 1990 to 2017: analysis for the Global Burden of Disease 2017 Study. | Different diagnostic criteria |
| Rosa - 2013 | Clinical, epidemiological and radiological of pneumonia in the elderly. Service Geriatric Hospital Almanzor Aguinaga Asenjo, July 2010-July 2011 | Different diagnostic criteria |
| Bensenor - 2012 | Household income, sex and respiratory mortality in Sao Paulo, Brazil, 1996-2010 | Different diagnostic criteria |
| Gimenes - 2012 | Respiratory pressures and expiratory peak flow rate of patients undergoing coronary artery bypass graft surgery | Different diagnostic criteria |
| Antunes - 2012 | Trends in hospitalizations for respiratory diseases in Salvador, Bahia State, Brazil, 1998-2009 | Different diagnostic criteria |
| Varela - 2012 | Variability in COPD: The PLATINO Study Viewpoint | Different publication type |
| Crawford - 2012 | Tobacco-Related Chronic Illnesses: A Public Health Concern for Jamaica | Different diagnostic criteria |
| Montes de Oca - 2011 | Paid employment in subjects with and without chronic obstructive pulmonary disease in five Latin American cities: the PLATINO study | Duplicated population |
| Washko - 2011 | Lung Volumes and Emphysema in Smokers with Interstitial Lung Abnormalities | Different country |
| Bensenor - 2011 | Chronic obstructive pulmonary disease in Brazil: mortality and hospitalization trends and rates, 1996-2008 | Different diagnostic criteria |
| Martin-Loeches - 2011 | Use of early corticosteroid therapy on ICU admission in patients affected by severe pandemic (H1N1)v influenza A infection | Different country |
| Volpe - 2011 | Association of chronic pulmonary obstructive disease (COPD) and complications in head and neck surgery | Different population |
| Silva - 2010 | Update on chronic obstructive pulmonary disease | Different publication type |
| Montes de Oca - 2010 | Acute bronchodilator responsiveness in subjects with and without airflow obstruction in five Latin American cities: The PLATINO study | Duplicated population |
| Arcanjo - 2018 | Clinical and laboratory characteristics associated with referral of hospitalized elderly to palliative care. | Different outcome |
| Souza - 2020 | Prevalence and factors associated with respiratory diseases and diarrhea in recyclable material cooperative workers in the city of SÃ£o Paulo, Brazil: a cross-sectional study, 2013 | Different diagnostic criteria |
| Fabra-Arrieta - 2019 | Respiratory disease associated with environmental and health factors in three villages from Guarne, Colombia, 2015 | Different diagnostic criteria |
| Kovelis - 2019 | Characteristics of long-term home oxygen therapy users in the municipality of Curitiba, Brazil | Different diagnostic criteria |
| Arancibia - 2017 | Chronic obstructive pulmonary disease and smoking | Different publication type |
| Signes-Costa - 2021 | Prevalence and 30-Day Mortality in Hospitalized Patients With Covid-19 and Prior Lung Diseases | Different diagnostic criteria |
| Evaristo-Mendez - 2016 | [Risk factors for nosocomial pneumonia in patients with abdominal surgery]. | Different diagnostic criteria |
| Fialkow - 2016 | Mechanical ventilation in patients in the intensive care unit of a general university hospital in southern Brazil: an epidemiological study | Different diagnostic criteria |
| CaÃ±ibano - 2020 | Prevalence of abdominal aortic aneurysms in a risk population seen in a vascular surgery department | Different diagnostic criteria |
| GarcÃ­a-Ortiz - 2020 | Evaluation with copd-ps questionnaire and vitalograph copd-6 portable device as a strategy for early diagnosis of copd in primary care | Different diagnostic criteria |
| Torre-Bouscoulet - 2015 | Comorbidities, sleep quality and quality of life in patients with lung cancer | Different diagnostic criteria |
| Llanqui - 2015 | Chemical irritants and prevalence of asthma and chronic bronchitis among cleaner workers of health institutions at the Puno region, Peru. | Different diagnostic criteria |
| Lizarbe - 2015 | Risk factors associated with Hospital complications in elderly patients to Hospital Nacional Edgardo Rebagliati Martins Lima, 2010 | Different diagnostic criteria |
| Sales - 2014 | Aortic Center: specialized care improves outcomes and decreases mortality | Different diagnostic criteria |
| Prudencio - 2014 | Epidemiology of COPD in Military Hospital | Different diagnostic criteria |
| Athanazio - 2012 | Airway disease: similarities and differences between asthma, COPD and bronchiectasis | Different study design |
| Teixeira - 2012 | Impact of a mechanical ventilation weaning protocol on the extubation failure rate in difficult-to-wean patients | Different diagnostic criteria |
| Lisanti - 2011 | Programa de oxigenoterapia domiciliaria de la Obra Social de Empleados públicos (OSEP), Mendoza (Argentina), experiencia de 7 años | Different diagnostic criteria |
| Sousa - 2011 | Prevalence of chronic obstructive pulmonary disease and risk factors in SÃ£o Paulo, Brazil, 2008-2009 | Different diagnostic criteria |
| Volpe - 2011 | Association of chronic pulmonary obstructive disease (COPD) and complications in head and neck surgery | Duplicate record |
| Souza - 2010 | Respiratory symptoms in charcoal production workers in the cities of Lindolfo Collor, Ivoti and Presidente Lucena, Brazil | Different diagnostic criteria |
| Mohammed - 2018 | A descriptive study of chronic obstructive pulmonary disease in tertiary care clinics of a Caribbean island | Different population |
| Horner - 2017 | Altitude and COPD prevalence: Analysis of the PREPOCOL-PLATINO-BOLD-EPI-SCAN study | Duplicated population |
| Jardim - 2017 | Respiratory medication use in primary careamongCOPDsubjects in four Latin American countries | Duplicated population |
| Vega - 2015 | Respiratory diseases in workers exposed to lateritic dust | Different diagnostic criteria |
| Watanabe - 2015 | Long term home oxygen therapy: Usersâ€™ profile and costs | Different outcome |
| Chen - 2014 | Native American ancestry, lung function, and COPD in Costa Ricans | Different study design |
| Dougados - 2014 | Prevalence of comorbidities in rheumatoid arthritis and evaluation of their monitoring: Results of an international, cross-sectional study (COMORA) | Different diagnostic criteria |
| Mendoza-Gonzalez - 2020 | EPIDEMIOLOGICAL ANALYSIS OF THE PANDEMIC BY SARS-COV2 IN MEXICAN POPULATION: EVALUATION OF MIDDLEWAY, CHARACTERISTICS, COMORBIDITY AND RISK | Different diagnostic criteria |
| Hernandez-Galdamez - 2020 | Increased Risk of Hospitalization and Death in Patients with COVID-19 and Pre-existing Noncommunicable Diseases and Modifiable Risk Factors in Mexico | Different diagnostic criteria |
| Tomaniak - 2020 | Impact of chronic obstructive pulmonary disease and dyspnoea on clinical outcomes in ticagrelor treated patients undergoing percutaneous coronary intervention in the randomized GLOBAL LEADERS trial | Different diagnostic criteria |
| Maas - 2020 | Prevalence of Chronic Bronchitis and Respiratory Health Profile of a Population Exposed to Wood Smoke in Nicaragua | Different diagnostic criteria |
| Goncalves-Macedo - 2019 | Trends in morbidity and mortality from COPD in Brazil, 2000 to 2016 | Different diagnostic criteria |
| Babanov - 2011 | Epidemiological characteristics of chronic obstructive pulmonary disease in the middle volga region | Different country |
| Krishnan - 2019 | Prevalence and Characteristics of Asthma-Chronic Obstructive Pulmonary Disease Overlap in Routine Primary Care Practices | Different country |
| Morgan - 2019 | Epidemiology and risk factors of asthma-chronic obstructive pulmonary disease overlap in low- and middle-income countries | Different study design |
| Valle - 2010 | Morbidity and mortality in patients aged over 75 years undergoing surgery for aortic valve replacement | Different diagnostic criteria |
| Carlos - 2010 | Association between indoor pollution, respiratory symptoms and COPD in Santiago, Chile: PLATINO Study | Duplicate record |
| De Vries - 2020 | Current state of respiratory disease in Suriname, a retrospective analysis of 4987 hospital files | Different diagnostic criteria |
| Trompeter - 2018 | Patterns of Body Composition Relating to Chronic Respiratory Diseases Among Adults in Four Resource-Poor Settings in Peru | Duplicated population |
| Freitas - 2017 | COPD Diagnosis Prevalence in Patients with Ischemic Heart Disease hospitalized in a university hospital in the interior of Rio Grande do Sul State | Different diagnostic criteria |
| Horner - 2016 | Altitude and COPD prevalence and other determinants: analysis of the PREPOCOL-PLATINO-BOLD-EPISCAN study | Duplicate record |
| Paschoalini - 2019 | Copd due to home biomass smoke exposure in rural non-smokers Brazilian women | Different publication type |
| Ruiz - 2018 | Assessing the burden of disease-associated malnutrition among hospitalized malnourished Colombian patients with heart and lung disease | Different population |
| Goebel - 2018 | Chronic obstructive pulmonary disorder in chronically exposed to silica: Experience of hospital Das clinicas da UFMG | Different diagnostic criteria |
| Oliveira - 2017 | Predictors of mediastinitis risk after coronary artery bypass surgery: Applicability of score in 1.322 cases | Different diagnostic criteria |
| Rodriguez - 2017 | Validation of the PUMA score for detecting COPD in a primary care population at the Hospital Maciel, Montevideo | Different publication type |
| Angarita - 2017 | COPD incidence in subjects with risk factors, chronic respiratory symptoms and normal spirometry: The PLATINO study> | Different publication type |
| Leslie - 2017 | Body composition as a risk of prolonged hospitalisations in patients with heart failure and / or respiratory distress | Different diagnostic criteria |
| Montes de Oca - 2017 | Asthma-COPD overlap syndrome (ACOS) in primary care of four Latin America countries: The PUMA study | Duplicated population |
| Montes de Oca - 2017 | Classification of patients with chronic obstructive pulmonary disease according to the Latin American Thoracic Association (ALAT) staging systems and the global initiative for chronic obstructive pulmonary disease (GOLD) | Duplicated population |
| Montes de Oca - 2017 | Classification of COPD Patients According to ALAT and GOLD Staging Systems Using PUMA Study Data | Duplicate record |
| Fernandez - 2017 | Tuberculosis in octogenarian patients between 2011-2016 in fundacion valle Del Lili, a reference Hospital In Latin America | Different diagnostic criteria |
| Guzman-Bouilloud - 2017 | Determining the prevalence Of COPD in primary care setting by using a telemedicine spirometry program. A screening study | Different publication type |
| de São José - 2016 | Primary care physicians' ability to diagnose the most prevalent respiratory diseases | Different diagnostic criteria |
| Torres-Duque - 2016 | Is Chronic Obstructive Pulmonary Disease Caused by Wood Smoke a Different Phenotype or a Different Entity? | Different study design |
| De Freitas - 2016 | Prevalence of asthma-chronic obstructive pulmonary disease (COPD) overlap syndrome in brazilian elderly patients | Different publication type |
| Bastidas - 2015 | Use of artificial intelligence in the diagnosis of chronic obstructive pulmonary disease (COPD) | Different study design |
| Torre-Bouscoulet - 2015 | Comorbidities, sleep quality and quality of life in patients with locally advanced lung cancer | Different diagnostic criteria |
| Antonio - 2015 | 48-hour fluid balance does not predict a successful spontaneous breathing trial | Different diagnostic criteria |
| Lopez - 2014 | Primary care COPD case finding in four latin america countries: The puma study | Different publication type |
| Casas - 2014 | Copd under-diagnosis and misdiagnosis in primary care population at high risk in four latin america countries: The puma study | Different publication type |
| Vianna - 2014 | Detection of COPD in a senior community center: High occurrence of undiagnosed COPD and reduced quality of life | Different publication type |
| Barbosa - 2014 | Chronic obstructive pulmonary disease (COPD)-searching the prevalence | Different publication type |
| Manzano - 2014 | Incidence of chronic obstructive pulmonary disease (COPD) based on three criteria for diagnosis: Follow-up Platino study-Sao Paulo, Brasil | Different publication type |
| Manzano - 2014 | Prospective analysis of smoking aspects over eight years in a population-based study-follow-up Platino study | Different publication type |
| Laniado - 2014 | Prevalence of chronic obstructive pulmonary disease in successfully treated pulmonary tuberculosis patients | Different population |
| Perez-Padilla - 2019 | Chronic Obstructive Pulmonary Disease in Latin America. | Duplicate record |
| Cherbuin - 2019 | Chronic Obstructive Pulmonary Disease and Risk of Dementia and Mortality in Lower to Middle Income Countries. | Different diagnostic criteria |
| Menezes - 2014 | CPOD and lung function parameters as predictors of mortality: Results from the Latin American PLATINO cohort study | Different publication type |
| Stelzer - 2014 | Chronic headache (CH) and obstructive sleep apnoea (OSA): Preliminary findings | Different diagnostic criteria |
| Menezes - 2014 | Increased risk of exacerbation and hospitalization in subjects with an overlap phenotype : COPD-Asthma | Duplicated population |
| Schiavi - 2014 | COPD screening in primary care in four latin American countries: Methodology of the PUMA study | Duplicated population |
| Hernández-Díaz - 2013 | Impact of coexisting COPD / smoking in the evolution of a cohort of patients lung carcinoma | Different diagnostic criteria |
| Martín - 2013 | Using the lower limit of normal (LLN) for the FEV1/fvc ratio changes the prevalence of airway obstruction in a hospital based-population | Different country |
| Angeli - 2019 | Detrimental Impact of Chronic Obstructive Pulmonary Disease in Atrial Fibrillation: New Insights from Umbria Atrial Fibrillation Registry. | Different country |
| Blanco - 2018 | Geographical Distribution of COPD Prevalence in the Americas. | Different study design |
| Grigsby - 2016 | Socioeconomic status and COPD among low- and middle-income countries. | Duplicated population |
| Kawassaki - 2013 | Differences in chest HRCT between smoking and non-smoking rheumatoid arthritis patients with abnormal spirometry | Different publication type |
| José - 2013 | The lack of awareness by general practitioners in diagnosing acute and chronic respiratory conditions in a low-income setting | Different diagnostic criteria |
| Morais - 2013 | Smoking status, prevalence of smoking-related diseases and their impact on costs in patients hospitalized in the thoracic units of a tertiary hospital in Brazil | Different diagnostic criteria |
| Nonato - 2013 | The frequency of number of respiratory symptoms in restrictive ventilatory impairment and chronic pulmonary obstructive disease related to disease severity: The Platino study | Different population |
| Torres-Duque - 2013 | Chronic obstructive pulmonary disease in people exposed to wood smoke. Prepocol: A population based study | Different publication type |
| de Carvalho - 2012 | Infrarenal abdominal aortic aneurysm: Significance of screening in patients of public hospitals in the metropolitan region of salvador - bahia, Brazil | Different diagnostic criteria |
| Roldan - 2012 | Lung cancer, chronic obstructive pulmonary disease (COPD) and tuberculosis in mexican women nonsmokers exposed to smoke from biomass | Different population |
| Loera - 2012 | Results of spirometry campaign hold between october 14 (world day spirometry) and november 17 (world COPD day) 2010 in Durango city, Mexico | Different publication type |
| Arbex - 2012 | Under-diagnosis of COPD in suspected lung cancer patients | Different publication type |
| Aaron - 2016 | Influence of country-level differences on COPD prevalence. | Different study design |
| Torres - 2018 | Trends in mortality from chronic obstructive pulmonary disease in Rio de Janeiro and Porto Alegre, Brazil, 1980-2014. | Different outcome |
| da Silva - 2012 | Impaired lung function in individuals chronically exposed to biomass combustion | Different outcome |
| Suzuki - 2011 | Estimating the budget impact of introducing indacaterol in the treatment of chronic obstructive pulmonary disease (COPD) from the public payer perspective in sÃ£o paulo | Different study design |
| Lutz - 2011 | Cost-utility analysis of varenicline vs existing smoking cessation strategies in EL Salvador | Different diagnostic criteria |
| Alvis - 2011 | Effect of biomass smoke on chronic obstructive pulmonary diseasein rural localities of Colombia | Different diagnostic criteria |
| Martins - 2011 | The prevalence of comorbidities in a cohort of chronic heart failure outpatients | Different diagnostic criteria |
| Machado - 2011 | Hospitalization rates due to chronic obstructive pulmonary disease and related costs before and after institution of outclinics for advanced lung diseases treatment in Sao Paulo City, Brazil (2004-2009) | Different study design |
| De Oliveira - 2011 | Alpha 1 antitrypsin deficiency in Brazil | Different population |
| Hernandez-Zenteno - 2011 | Dyspnea is a good predictor for early diagnosis of COPD. A comparison of a symptoms-based versus a non-symptoms based strategies to detect smokers with COPD | Different publication type |
| Cabrera-Serrano - 2019 | Tobacco Use and Associated Health Conditions and Risk Factors in the Lesbian, Gay, Bisexual, Transgender, and Transsexual Populations of Puerto Rico, 2013-2015. | Different diagnostic criteria |
| Santos - 2014 | Characteristics of undiagnosed COPD in a senior community center. | Different diagnostic criteria |
| Perez-Padilla - 2012 | Airflow obstruction in never smokers in five Latin American cities: the PLATINO study. | Duplicated population |
| Laniado-Laborin - 2010 | The prevalence of airway obstruction in subjects with a history of pulmonary tuberculosis in Mexico | Different outcome |
| Estenssoro - 2010 | Epidemiology, risk factors, clinical characteristics and oseltamivir use in adult patients admitted to the ICU with acute respiratory failure requiring mechanical ventilation during 2009 Influenza A H1N1 Pandemia | Different diagnostic criteria |
| Estenssoro - 2010 | Mechanical ventilation in adult patients admitted to the ICU with acute respiratory failure during 2009 influenza A H1N1 pandemia: Are There differences between confirmed vs. probable and suspected cases? the registry of the argentinean society of intensive care medicine (SATI) | Different diagnostic criteria |
| Silva - 2010 | Prevalence of patients with respiratory symptoms as a primary reason for seeking an outpatient unit and an emergence service in rio de janeiro, Brazil-preliminary results | Different publication type |
| Ugarte - 2010 | Influenza A pandemics: Clinical and organizational aspects: The experience in Chile | Different diagnostic criteria |
| de Souza - 2010 | Respiratory symptoms in charcoal production workers in the cities of lindolfo collor, ivoti and presidente lucena, Brazil | Duplicate record |
| Sousa - 2011 | Prevalence of chronic obstructive pulmonary disease and risk factors in SÃ£o Paulo, Brazil, 2008-2009. | Different diagnostic criteria |
| Landis - 2014 | Continuing to Confront COPD International Patient Survey: methods, COPD prevalence, and disease burden in 2012-2013. | Different diagnostic criteria |
| Moreira - 2014 | PLATINO, a nine-year follow-up study of COPD in the city of SÃ£o Paulo, Brazil: the problem of underdiagnosis. | Duplicated population |
| Lopez - 2010 | Sex-related differences in COPD in five Latin American cities: the PLATINO study. | Duplicated population |
| Menezes - 2014 | A population-based cohort study on chronic obstructive pulmonary disease in Latin America: methods and preliminary results. The PLATINO Study Phase II. | Different diagnostic criteria |
| Rubinstein - 2011 | Detection and follow-up of chronic obstructive pulmonary disease (COPD) and risk factors in the Southern Cone of Latin America: the pulmonary risk in South America (PRISA) study. | Different publication type |
| Olmos - 2015 | [Primary care consultations due to respiratory diseases in the period 2003-2008]. | Different diagnostic criteria |
| Guarnieri - 2015 | Lung Function in Rural Guatemalan Women Before and After a Chimney Stove Intervention to Reduce Wood Smoke Exposure: Results From the Randomized Exposure Study of Pollution Indoors and Respiratory Effects and Chronic Respiratory Effects of Early Childhood Exposure to Respirable Particulate Matter Study. | Different outcome |
| Lasa - 2012 | [Bacteremia in patients hospitalized with cellulitis]. | Different diagnostic criteria |
| Pérez - 2011 | Preoperative clinical and demographic characteristis in Puerto Rico patients referred to inpatient cardiac rehabiliation after cardiac surgery. | Different diagnostic criteria |
| Denktaş - 2010 | Underutilization of prescribed drugs use among first generation elderly immigrants in the Netherlands. | Different country |
| Queiroz - 2012 | Underdiagnosis of COPD at primary health care clinics in the city of Aparecida de GoiÃ¢nia, Brazil. | Duplicate record |
| Zaitune - 2012 | [Factors associated with smoking in the elderly: a health survey in SÃ£o Paulo (ISA-SP)]. | Different diagnostic criteria |
| Teixeira - 2012 | Impact of a mechanical ventilation weaning protocol on the extubation failure rate in difficult-to-wean patients. | Duplicate record |
| Moreira - 2015 | Prevalence of Pulmonary Hypertension in the General Population: The Rotterdam Study. | Different country |
| Macedo-Viñas - 2018 | Estimating the Burden of Serious Fungal Infections in Uruguay. | Different diagnostic criteria |
| Camacho - 2020 | Self-Reported Prevalence of Chronic Non-Communicable Diseases in Relation to Socioeconomic and Educational Factors in Colombia: A Community-Based Study in 11 Departments. | Different diagnostic criteria |
| Fabra-Arrieta - 2019 | [Respiratory disease associated with environmental and health factors in three villages from Guarne, Colombia, 2015]. | Duplicate record |
| Comim - 2017 | Higher prevalence of clinical cardiovascular comorbidities in postmenopausal women with self-reported premenopausal hirsutism and/or oligo-amenorrhea. | Different diagnostic criteria |
| Flores - 2010 | Association between indoor pollution, respiratory symptoms and COPD in Santiago, Chile: PLATINO Study | Duplicated population |
| Sansores - 2013 | Prevalence and diagnosis of chronic obstructive pulmonary disease among smokers at risk. A comparative study of case-finding vs. screening strategies | Duplicated population |
| Laniado-Laborin - 2012 | High altitude and chronic obstructive pulmonary disease prevalence: a casual or causal correlation? | Duplicated population |
| Lamprecht - 2015 | Determinants of underdiagnosis of COPD in national and international surveys | Different diagnostic criteria |
| Pilla – 2018 | Rhinosinusitis symptoms, smoking and COPD: Prevalence and associations | Duplicate population |

| Supplementary material 3. Characteristics of the included study assessing the incidence of chronic obstructive pulmonary disease in Latin America and the Caribbean (n=1). | | | | | | | | | |
| --- | --- | --- | --- | --- | --- | --- | --- | --- | --- |
| Study id | Country | Setting | Follow-up | Smoking status | n | Age (years) Mean ± SD | Male sex (%) | COPD cumulative incidence (%) | Quality score (Max. 9) |
| Laender - 2015 | Brazil | General population | 9 years | Non-smokers, ex-smokers or smokers | 594 | 62.4 ± 9.9 | 45.5 | 3.4 | 8 |
| COPD: Chronic obstructive pulmonary disease. | | | | | | | | | |

Supplementary material 4. Prevalence of chronic obstructive pulmonary disease by sex in the general population of Latin America and the Caribbean.


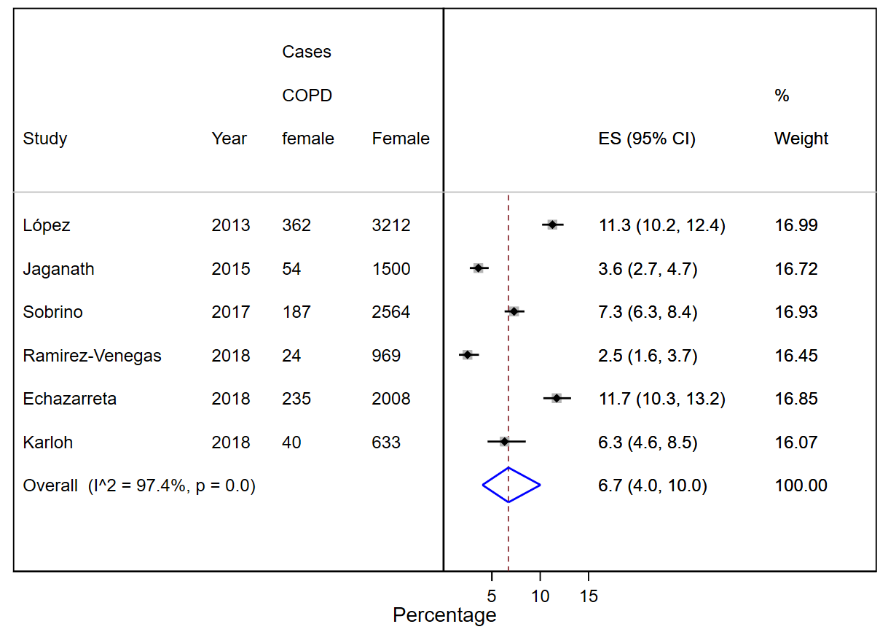

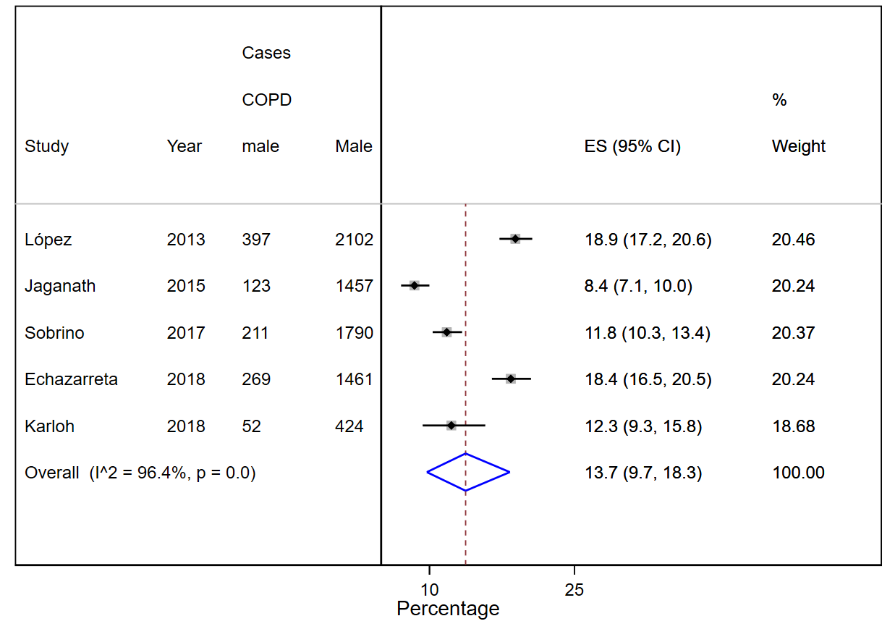
 Male Female

Supplementary material 5. Prevalence of chronic obstructive pulmonary disease in the general population of Latin America and the Caribbean by countries.


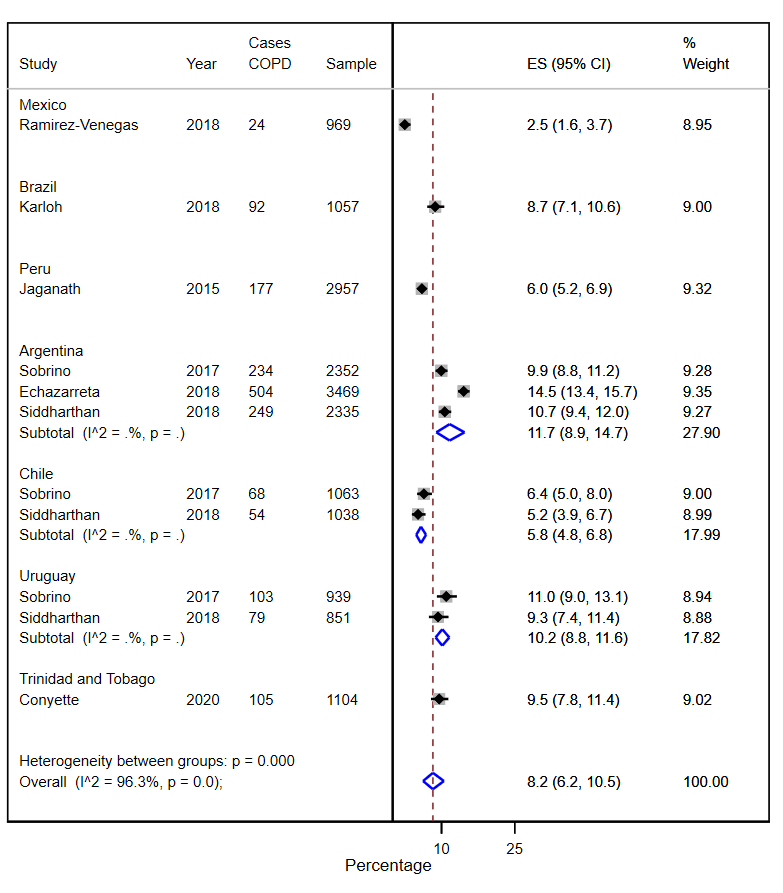


Supplementary material 6. Prevalence of chronic obstructive pulmonary disease by risk of bias score (higher score, higher methodological quality) in Latin America and the Caribbean.


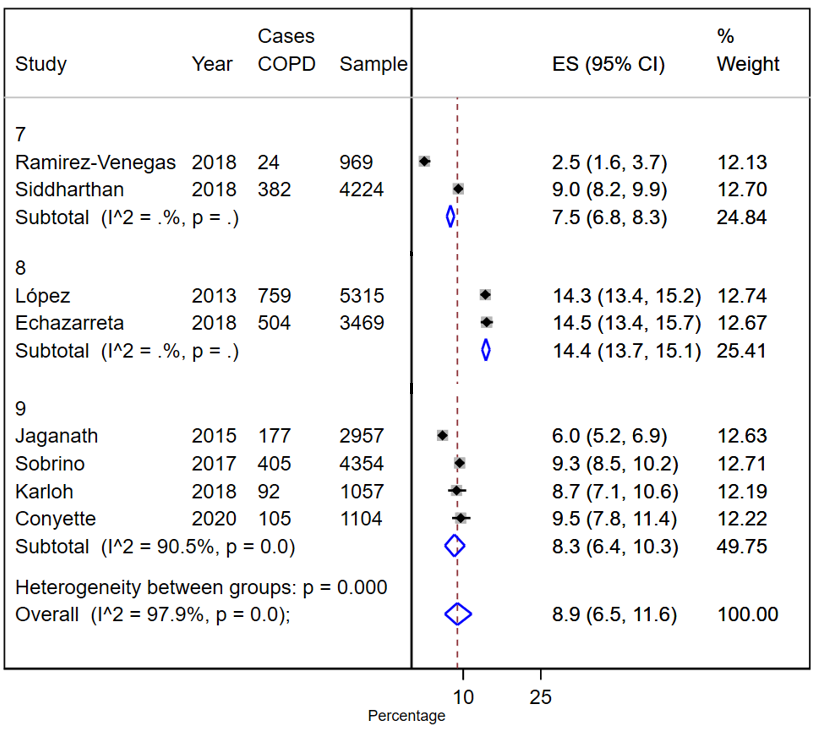


Supplementary material 7. Prevalence of chronic obstructive pulmonary disease in smokers and ex-smokers of Latin America and the Caribbean.


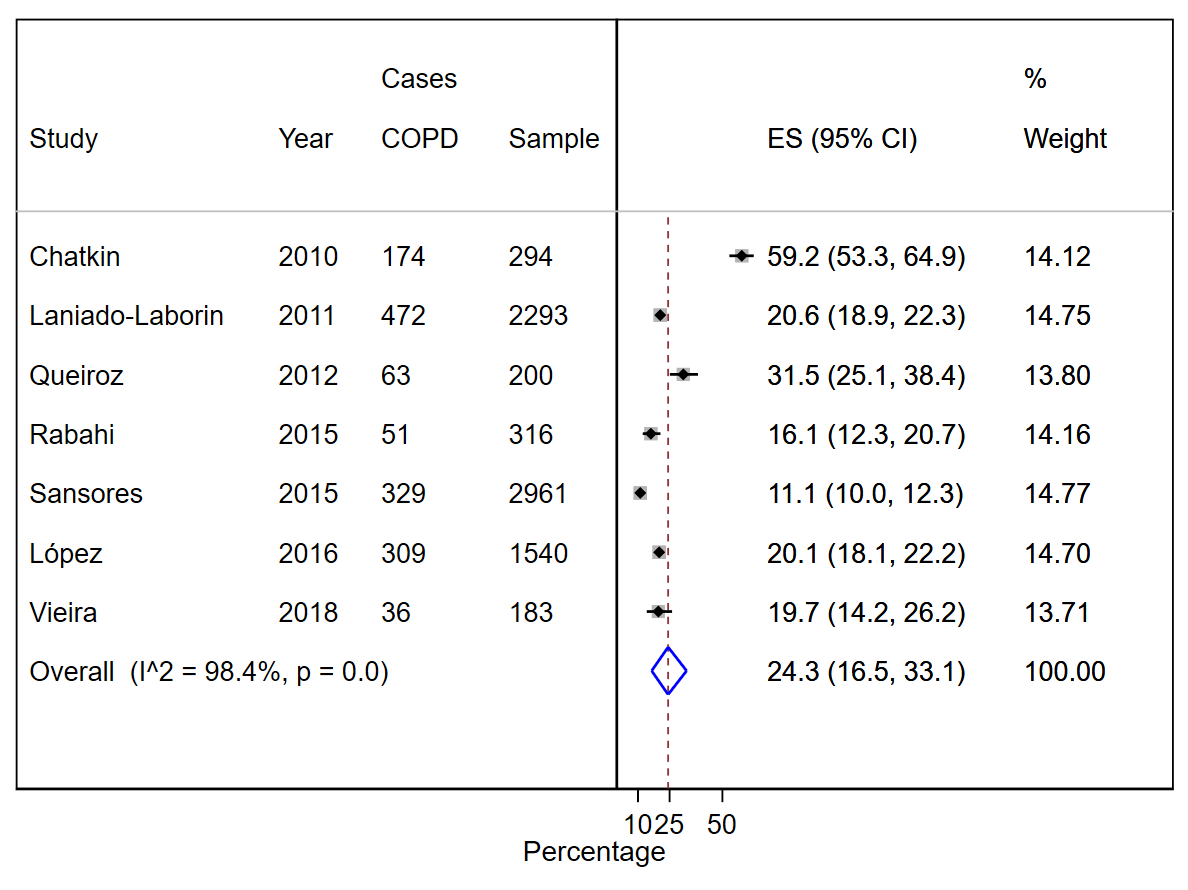


Supplementary material 8. Prevalence of chronic obstructive pulmonary disease by setting in Latin America and the Caribbean. Sensitivity analysis of the variation in prevalence if each included study is removed.


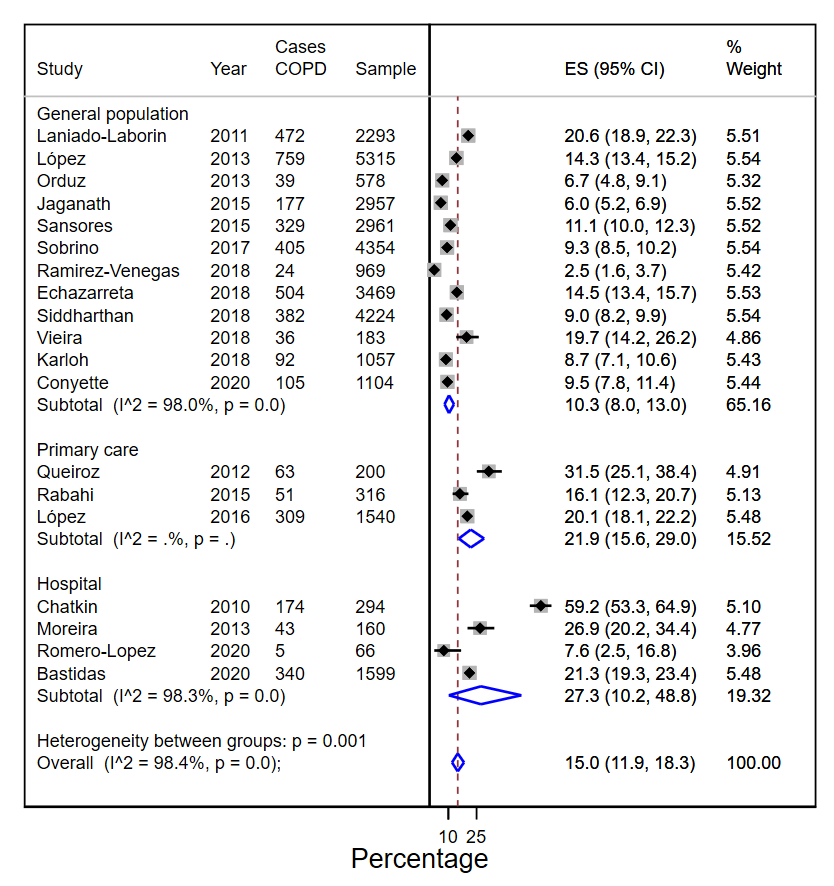


| Supplementary material 9. Risk of bias of included studies using the Joanna Briggs Institute Critical Apraissal Tool for prevalence studies. | | | | | | | | | |
| --- | --- | --- | --- | --- | --- | --- | --- | --- | --- |
| Study id | Was the sample frame appropriate to address the target population? | Were study participants sampled in an appropriate way? | Was the sample size adequate? | Were the study subjects and the setting described in detail? | Was the data analysis conducted with sufficient coverage of the identified sample? | Were valid methods used for the identification of the condition? | Was the condition measured in a standard, reliable way for all participants? | Was there appropriate statistical analysis? | Was the response rate adequate, and if not, was the low response rate managed appropriately? |
| Romero-Lopez - 2020 | No | Unclear | No | Yes | No | Yes | Yes | Yes | Yes |
| Bastidas - 2020 | No | No | Yes | Yes | Yes | Yes | Yes | No | Yes |
| Conyette - 2020 | Yes | Yes | Yes | Yes | Yes | Yes | Yes | Yes | Yes |
| Ramirez-Venegas - 2018 | Yes | Yes | Yes | Yes | No | Yes | Yes | No | Yes |
| Pilla - 2018 | Yes | Yes | Yes | Yes | Yes | Yes | Yes | No | Yes |
| Echazarreta - 2018 | Yes | Yes | Yes | Yes | Yes | Yes | Yes | Yes | No |
| Yesddharthan - 2018 | Yes | Yes | Yes | Yes | Yes | Yes | Yes | No | Unclear |
| Vieira - 2018 | No | Yes | Yes | Yes | Yes | Yes | Yes | No | No |
| Karloh - 2018 | Yes | Yes | Yes | Yes | Yes | Yes | Yes | Yes | Yes |
| SobriNo - 2017 | Yes | Yes | Yes | Yes | Yes | Yes | Yes | Yes | Yes |
| López - 2016 | No | No | Yes | Yes | Yes | Yes | Yes | No | Yes |
| Jaganath - 2015 | Yes | Yes | Yes | Yes | Yes | Yes | Yes | Yes | Yes |
| Laender - 2015 | Yes | Yes | Yes | Yes | Yes | Yes | Yes | Yes | No |
| Rabahi - 2015 | No | No | Yes | No | Unclear | Yes | Yes | No | Unclear |
| Sansores - 2015 | No | No | Yes | Yes | Yes | Yes | Yes | No | Yes |
| López - 2013 | Yes | Yes | Yes | Yes | Yes | Yes | Yes | No | Yes |
| Moreira - 2013 | No | Unclear | No | Yes | No | Yes | Yes | No | Unclear |
| Orduz - 2013 | Yes | Yes | Yes | No | Unclear | Yes | Yes | No | Unclear |
| Queiroz - 2012 | No | No | Yes | Yes | Yes | Yes | Yes | No | Yes |
| Laniado-Laborin - 2011 | Yes | No | Yes | Yes | Yes | Yes | Yes | Yes | Yes |
| Chatkin - 2010 | No | No | Yes | Yes | Yes | Yes | Yes | No | Unclear |
